# Synthesis, Characterization, and Physical Properties of Maleic Acid-Grafted Poly(butylene adipate-co-terephthalate)/Cellulose Nanocrystal Composites

**DOI:** 10.3390/polym14132742

**Published:** 2022-07-05

**Authors:** Yu-Jia Hung, Ming-Yen Chiang, En-Tze Wang, Tzong-Ming Wu

**Affiliations:** Department of Materials Science and Engineering, National Chung Hsing University, 250 Kuo Kuang Road, Taichung 402, Taiwan; tvickytw@yahoo.com.tw (Y.-J.H.); tigercity86@gmail.com (M.-Y.C.); axsz2760089@gmail.com (E.-T.W.)

**Keywords:** biodegradable copolyesters, composites, mechanical property, water vapor permeation

## Abstract

New sequences of nanocomposites including numerous maleic acid-grafted poly(butylene adipate-co-terephthalate) (g-PBAT) and cellulose nanocrystals (CNC) were efficaciously fabricated via transesterification and polycondensation processes with the covalent bonds between the polymer and reinforcing fillers. The grafting interaction of maleic acid onto PBAT was successfully demonstrated using Fourier transform infrared (FTIR) and ^13^C-nuclear magnetic resonance (NMR) spectra. The morphology of g-PBAT/CNC nanocomposites was investigated by wide-angle X-ray diffraction and transmission electron microscopy. Both results indicate that the CNC was randomly dispersed into the g-PBAT polymer matrix. The storage modulus at −80 and 25 °C was significantly enhanced with the incorporation of CNC into g-PBAT matrix. The crystallization rate of g-PBAT/CNC nanocomposites increased as the loading of CNC increased. With the incorporation of 3 wt% CNC, the half-time for crystallization of the g-PBAT/CNC composite decreased about 50~80% as compared with the same isothermal crystallization of pure polymer matrix. All water vapor permeation (WVP) values of all g-PBAT/CNC nanocomposites decreased as the loading of CNC increased. The decrease in WVP may be attributed to the addition of stiff CNC, causing the increase on the permeation route in the water molecules in the g-PBAT polymer matrix.

## 1. Introduction

Biodegradable copolyesters with aliphatic–aromatic conformation have received significant attention because of the tremendous biodegradable properties of their aliphatic components and outstanding mechanical properties of the aromatic components around their polymer backbones [1,2,3]. Among these important copolymers, biodegradable poly(butylene adipate-co-terephthalate) (PBAT) is prepared by transesterification and polycondensation of 1,4-butanediol (BD) with adipic acid (AA) and dimethylene terephthalate (DMT) or terephthalic acid (TPA) [4,5,6]. Owing to the existence of the aromatic component of DMT or TPA, PBAT has been extensively studied because of its exceptional physical properties as compared to another biodegradable poly[(butylene succinate)-co-adipate] (PBSA) [7,8]. To further enhance its physical properties, the addition of inorganic material, including layered silicates and layered double hydroxides, can serve to strengthen PBAT [9,10,11,12,13]. Wu et al. examined the effect of adding layered zinc phenylphosphonate (PPZn) on the mechanical properties, thermal stability, and degradation behaviors of PBAT. The storage modulus and degradation rate of PBAT/PPZn nanocomposites are significantly enhanced by adding PPZn into PBAT matrix. Moustafa et al. confirmed that the PBAT composites could be prospective candidates used in the applications of food packaging [14].

Cellulose nanocrystals (CNCs) containing excellent mechanical properties, such as axial elastic modulus and high stiffness, have recently been developed [15,16,17]. In addition, several previous investigations have proved that they also display exceptional biocompatibility [18,19,20,21]. Owing to their exceptional mechanical and biodegradable properties, the incorporation of CNC into biodegradable polymers has received much attention [22,23,24,25,26,27]. Shojaeiarani et al. investigated the rheological properties of poly(lactic acid) (PLA)/CNC composites [23]. Their data revealed that a network-like structure was formed in the composites, which was due to the presence of the CNC restricting the flow of PLA chains in the molten state. Qiu et al. investigated the crystallization behaviors of PBSA/CNC composites [24]. Their results exhibited that the presence of CNC served as an efficient nucleating agent, enhancing the crystallization rates of the composites. Based on the above studies, the interaction between biodegradable polymer and CNC is noncovalent bonding. Normally, covalent bonds between polymer and supporting material are more stable and useful than noncovalent bonds. However, no report on PBAT/CNC composites with covalent linkages between polymer and supporting material has been made. In order to extend the applications of PBAT, the study of PBAT/CNC composites with covalent linkages between PBAT and CNC is necessary. Therefore, the chemical modification of PBAT is necessitated to provide the formation of covalent linkages with the supporting CNC material.

In this study, a series of maleic acid-grafted poly(butylene adipate-co-terephthalate) (g-PBAT)/cellulose nanocrystals (CNCs) nanocomposites with covalent bonding between the polymer and supporting materials were effectively synthesized. To our knowledge, this is the first report of g-PBAT/CNC nanocomposites with covalent bonding between g-PBAT and CNC. The mechanical and water vapor permeation (WVP) of g-PBAT/CNC nanocomposites were examined thoroughly.

## 2. Experimental

### 2.1. Materials

Adipate acid (AA), maleic acid (MA), microcrystalline cellulose ((C_6_H_10_O_5_)n, MCC), 2,2,6,6-tetramethylpiperidine-1-oxyl (C_9_H_18_NO, TEMPO), and sodium bromide (NaBr) were acquired from Sigma-Aldrich (St. Louis, MO, USA). Benzoyl peroxide (C_14_H_10_O_4_), sodium hydroxide (NaOH), sodium hypochlorite (NaClO), and hydrochloric acid (HCl) were purchased from Fluka Chemical Company (Charlotte, NC, USA). 1,4-butanediol (C_4_H_10_O_2_, BD), dimethylene terephthalate (C_10_H_10_O_4_, DMT), 1,12-dodecanediamine, tetrabutyl titanate (C_16_H_36_O_4_Ti), and 1-ethyl-3-(3-dimethylaminopropyl)carbodiimide (C_8_H_17_N_3_·HCl, EDC) were obtained from Alfa Aesar Chemical Company (Ward Hill, MA, USA). All chemicals were utilized as received.

### 2.2. Fabrication of g-PBAT/CNC Nanocomposites

**Figure 1:**
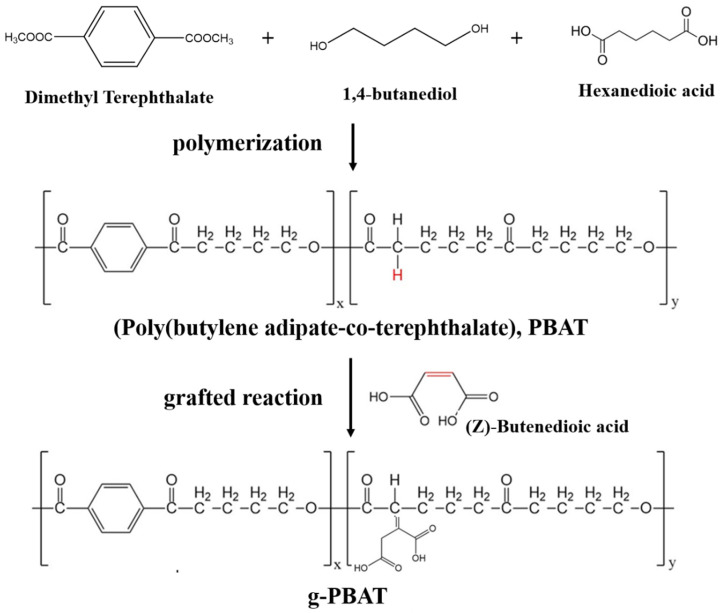
The reaction scheme of synthesis and grafted interaction of PBAT.

The reaction scheme of synthesis and grafted interaction of PBAT are shown in Figure 1. Three molar ratios of PBAT were manufactured via transesterification and polycondensation using the approach reported in previous literature [10,11]. Briefly, selected contents of AA, BD, DMT, and tetrabutyl titanate used as a catalyst were heated for 1 h at 160 °C and subsequently heated to 190 °C for 2 h to completely distill methanol and water. Finally, the obtained materials were heated to 220 °C for 4 h under vacuum. The feed molar ratios of [AA] to [DMT] were 40:60, 60:40, and 80:20 and desired contents of BD; the resulting materials are assigned as PBAT-40, PBAT-60, and PBAT-80, respectively. The synthesized polymer ratio determined using ^1^H-nuclear magnetic resonance is shown in Table 1.

The obtained PBAT and maleic acid were individually and homogeneous dissolved in chloroform and acetone. When chloroform and acetone are mixed together, a hydrogen bond is formed between them, which increases the interatomic interaction. Therefore, both solutions were then mixed and mechanically stirred for 3 h at 60 °C. The benzoyl peroxide dissolved in chloroform was added to the prepared solution for 24 h at 60 °C to allow the occurrence of grafting reaction (henceforth assigned as g-PBAT). The cellulose nanocrystals (CNCs) were fabricated from microcrystalline cellulose (MCC) according to previous reports [28,29]. Various quantities of g-PBAT, CNC, and 1-ethyl-3-(3-dimethylaminopropyl)carbodiimide (EDC) used as catalyst were exclusively dissolved in dichloromethane and then mechanically mixed and stirred for 3 days. The prepared g-PBAT/CNC nanocomposites were washed and dried under vacuum.

### 2.3. Analytical Procedures

The PerkinElmer Pyris Diamond differential scanning calorimeter (DSC, Waltham, MA, USA) was applied to evaluate the isothermal crystallization behavior of nanocomposites. All measurements were obtained under nitrogen environment. These experiments were heated at a rate of 10 °C/min to the designed temperatures (T_ds_), which are approximately 40 °C higher than the melting temperatures of g-PBAT, and maintained for 5 min to remove the remaining crystals. Consequently, the samples were immediately cooled to desired isothermal crystallization temperatures (T_cs_) and maintained to entirely complete the isothermal crystallization. The isothermal crystallization kinetics evaluated by Avrami equation could be clarified as follows: [30,31]

(1) $$1-X_{t}=\exp(-kt^{n})$$

where *X_t_* is the relative degree of crystallinity at crystallization time *t*, *n* is the Avrami exponent, and *k* is the crystallization rate constant. Equation (1) can be conveyed into its natural logarithm formula and shown in this way:

(2) $$\ln\left[-\ln\left(1-X_{t}\right)\right]=n\;\ln t+\ln k$$

The time at *X_t_* is equal to 50%, defined as the half-time of crystallization (*t*_1/2_), which is recognized using Equation (3).

(3) $$t_{1/2}={\left(\frac{\ln2}{k}\right)}^{1/n}$$

The thermal behaviors of samples were measured using TGA 2950 thermal gravimetric analyzer (TA Instruments, New Castle, DE, USA). These measurements were obtained under a nitrogen environment from room temperature to 800 °C at a heating rate of 10 °C/min. X-ray diffractometer (Bruker D8, Karlsruhe, Germany) equipped with a Ni-filtered Cu Kα radiation source was used for the experiments with wide-angle X-ray diffraction (WAXD). The diffraction profiles were observed ranging from 2θ = 1.5°–30° at a scanning rate of 1°/min. The transmission electron microscopy (TEM) images were obtained using JEOL JEM-2010 (Tokyo, Japan). TEM samples were made using a Reichert Ultracut ultramicrotome. Fourier transform infrared (FTIR) measurements were obtained under a PerkinElmer Spectrum One spectrometer (Waltham, MA, USA) in a range of 400–4000 cm^−1^.

Both ^1^H-nuclear magnetic resonance (NMR) and ^13^C-NMR spectra were carried out using Agilet Technologies DD2 600MHz NMR spectroscopy (Santa Clara, CA, USA) via CDCl_3_ as solvent and internal standard. The weight-average molecular weight (M_w_), number-average molecular weight (M_n_), and polydispersity PDI = M_w_/M_n_ of the prepared materials were established using gel-permeation chromatography (GPC; Waters 717 Plusautosampler, Waters Instruments, Rochester, NY, USA). The PerkinElmer dynamic mechanical analyzer (DMA8000, Waltham, MA, USA) was used to obtain the storage modulus (E’) from −80 to 40 °C at a heating rate of 2 °C/min and 1 Hz constant frequency.

Water vapor barrier property of nanocomposites determined by water vapor transmission rate (WVTR) was carried out using a Permatran-W Model 3/61 water vapor permeability meter (Ametek Mocon, Minnesota, MN, USA). The dimensions of test samples were 5 cm × 5 cm. The experiments were performed at 25 °C with relative humidity of 100%. The values of water vapor permeability (WVP) were estimated using the following equation: WVP = WVTR × D/ΔP, where D is the test sample thickness and ΔP = S x (RH_2_ – RH_1_). In the formula, S is saturated water vapor pressure, and RH_1_ and RH_2_ are the relative humidity of top and bottom test chambers, respectively.

## 3. Results and Discussion

### 3.1. Preparation and Characterization of CNCs

**Figure 2:**
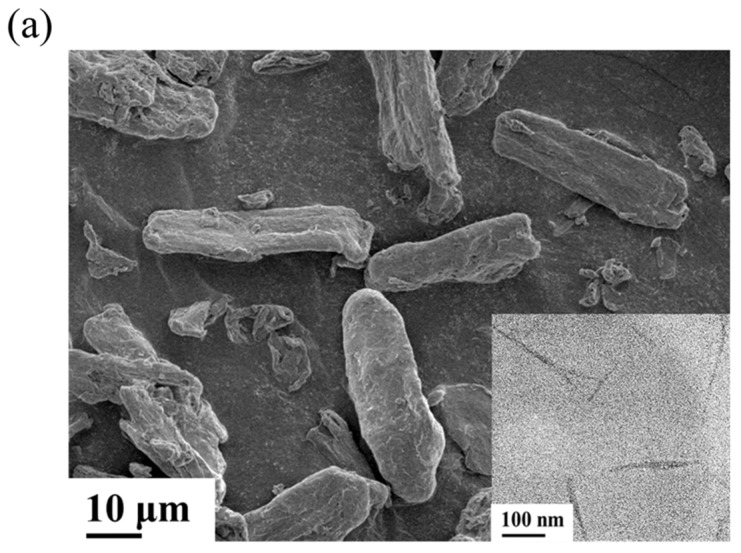

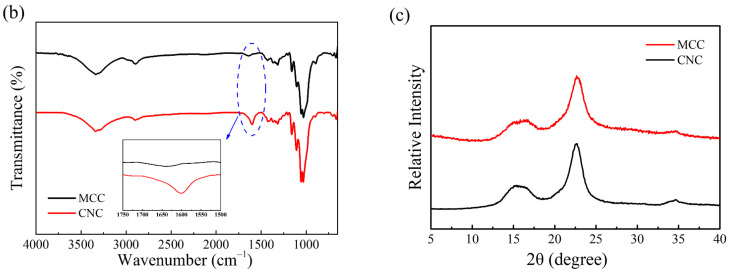
(**a**) SEM and TEM (insert) images, (**b**) FTIR spectrum, and (**c**) WAXD patterns of CNC and MCC.

TEMPO-mediated oxidation, a surface effect in which anionic carboxylate groups are introduced as functional groups at surfaces, is a useful technique to produce cellulose nanomaterial from its natural state [28,29]. Figure 2a presents a SEM image of the MCC, and the insert TEM image in Figure 2a is CNC. These images obviously exhibit that the dimensions of the MCC were significantly decreased to about hundreds of nanometers in length and less than 20 nm in width after TEMPO-mediated oxidation. The average length and width of the CNC were 155.5 ± 29.6 nm and 18.6 ± 3.3 nm, respectively. Figure 2b shows the FTIR spectrum of the MCC and CNC. The absorption peaks of MCC at 3200–3400 cm^−1^ are assigned to the free O-H stretching modes of the OH groups. The characteristic peaks at 2900 and 1425 cm^−1^ correspond to the CH_2_ stretching and symmetric bending vibration, respectively. The absorption peaks at 1160, 1105, 1055, and 1030 cm^−1^ are due to the glucosidic linkages of the cellulose chain. The peak at 897 cm^−1^ is related to the CH deformation vibration of unit rings [29,32]. There was a weak absorption peak at 1643 cm^−1^, which was attributed to the O-H bending vibration of the absorbed water [33,34]. After the TEMPO-mediated oxidation, an apparent difference can be seen in the FTIR spectrum of CNC exhibited in Figure 2b. A new absorption peak at 1604 cm^−1^ is assigned to the primary hydroxyls of alcohol changed into carboxyl groups on the surface of cellulose [28,29]. The WAXD patterns of the MCC and CNC presented in Figure 2c are almost identical. Both diffraction patterns of MCC and CNC exhibit the crystal phase related to the cellulose I crystal structures [29,32]. This result reveals that the chemical modification of MCC using TEMPO-mediated oxidation did not alter the crystal structure of the MCC.

### 3.2. Crystalline Structure and Molecular Weight of g-PBAT

**Figure 3:**
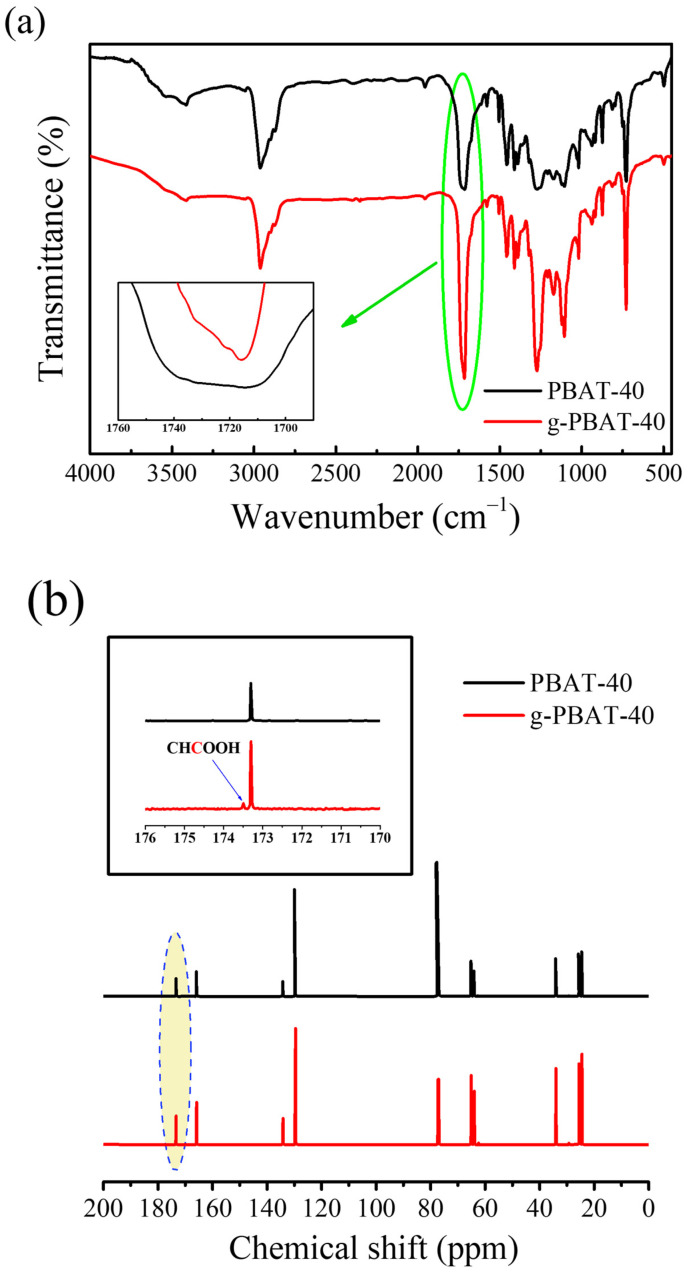
(**a**) FTIR and (**b**) ^13^C–NMR spectrum of the PBAT-40 and g-PBAT-40 copolyesters.

**Figure 4:**
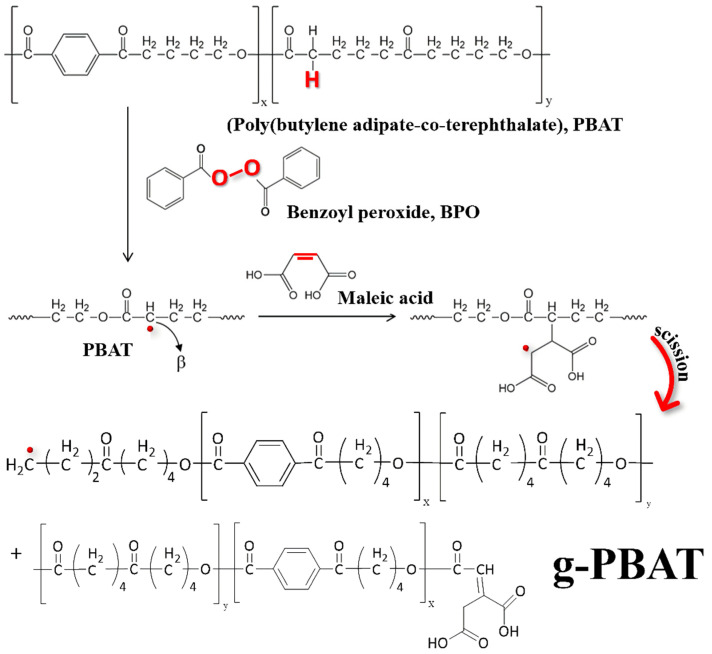
The possible mechanism of the free-radical reactions for the PBAT copolyester.

The FTIR spectra of PBAT-40 and g-PBAT-40 are shown in Figure 3a. The absorption peak of PBAT-40 and g-PBAT-40 at 1010 cm^−1^ is assigned to the O-C-C bond stretching vibration in the polymer [9,10]. The characteristic absorption peaks at about 1093 and 1274 cm^−1^ correspond to the –COC- bond stretching vibration in the ester group. The absorption peak at 1716 cm^−1^ is assigned to the stretching vibration of the PBA ester group. The absorption bands at 1400 and 1454 cm^−1^ are related to the trans-CH_2_-plane bending vibration. An additional peak of g-PBAT-40 at 1722 cm^−1^ due to –C=O is obtained, which reveals the existence of free acid in the chemically modified PBAT. This result proves the grafting interaction of maleic acid (MA) onto PBAT was successful. Similar results of grafting interaction by acrylic acid were investigated in previous reports [8,35]. More support for the grafting formation of MA using the ^13^C-NMR spectra is exhibited in Figure 3b. Both ^13^C-NMR spectra of PBAT-40 and g-PBAT-40 are analogous, except for an additional peak at δ = 173.5 ppm. This peak is attributed to the C=O bond of MA, which is confirmed by the grafting of MA onto PBAT [9,10]. The molecular weights of the various g-PBAT copolymers determined using GPC are given in Table 2. The M_n_ and PDI of prepared g-PBAT copolymer are in the ranges of 11,400~34,400 g/mol and 1.94~2.40, respectively, which are significantly different from pure polymer matrix. The change in molecular weight that occurred during the grafting reaction might be due to the occurrence of free-radical reactions. Figure 4 illustrates the possible mechanism of the free-radical reactions for the PBAT copolyester. Because the of non-propagation nature of the monomer, the peroxide initiators degraded at certain temperatures, and free radicals were generated. Then, the hydrogen atoms bonded with β carbon of the ester bond in PBAT polymer chains were extracted via cumyl or benzoyl radicals, whereas free radicals were generated on polymer chains. In the meantime, chain scission might occur owing to the instability of free radicals [36]. The melting temperatures of g-PBAT determined by DSC are 156.7 °C, 106.4 °C, and 42.8 °C for g-PBAT-40, g-PBAT-60, and g-PBAT-80, respectively. The melting temperatures of g-PBAT were slightly higher than pure PBAT, which may be attributed to the more crystallite chain packing during the crystallization formation.

**Figure 5:**
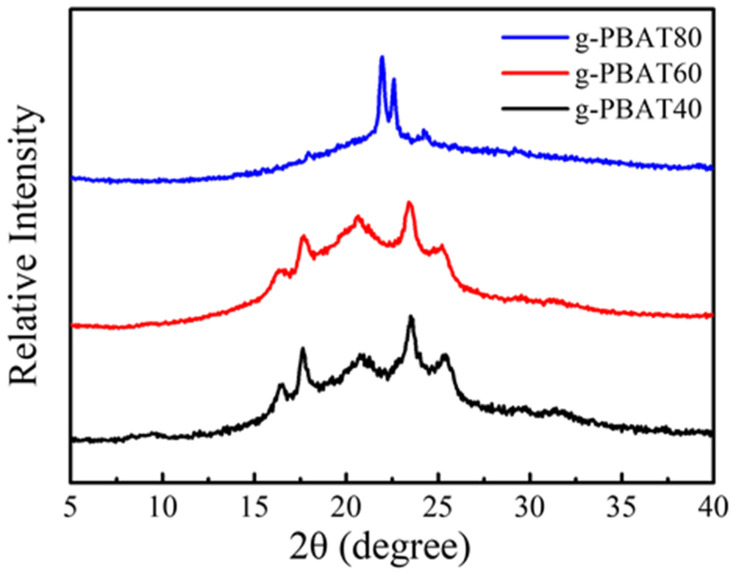
WAXD data for the g-PBAT-40, g-PBAT-60, and g-PBAT-80 copolyesters.

The WAXD diffraction curves of various compositions of g-PBAT copolyesters are shown in Figure 5. For the g-PBAT copolymers comprising two crystallizable comonomer components, the crystalline structure of g-PBAT clearly depends on the compositions of copolyesters. For the g-PBAT-40 and g-PBAT-60, the WAXD data reveal five major diffraction peaks at 2θ = 16.3°, 17.4°, 20.6°, 23.3°, and 25.3°, which are found to be in accordance with those of PBT crystallite [3,4]. These results prove that the crystalline structures of g-PBAT-40 and g-PBAT-60 are controlled by the PBT crystallite. The diffraction curve of the g-PBAT-80 copolymer also shown in this figure is entirely different from those of g-PBAT-40 and g-PBAT-60. Three strong diffraction peaks at 2θ = 21.8°, 22.4°, and 24.0° obtained are assigned to the PBA crystallite [4]. This experimental result discloses that the fabricated g-PBAT-80 copolymer is changed from the PBT crystalline structure into the PBA crystalline structure.

### 3.3. Structure, Morphology, and Physical Properties of g-PBAT/CNC Nanocomposites

**Figure 6:**
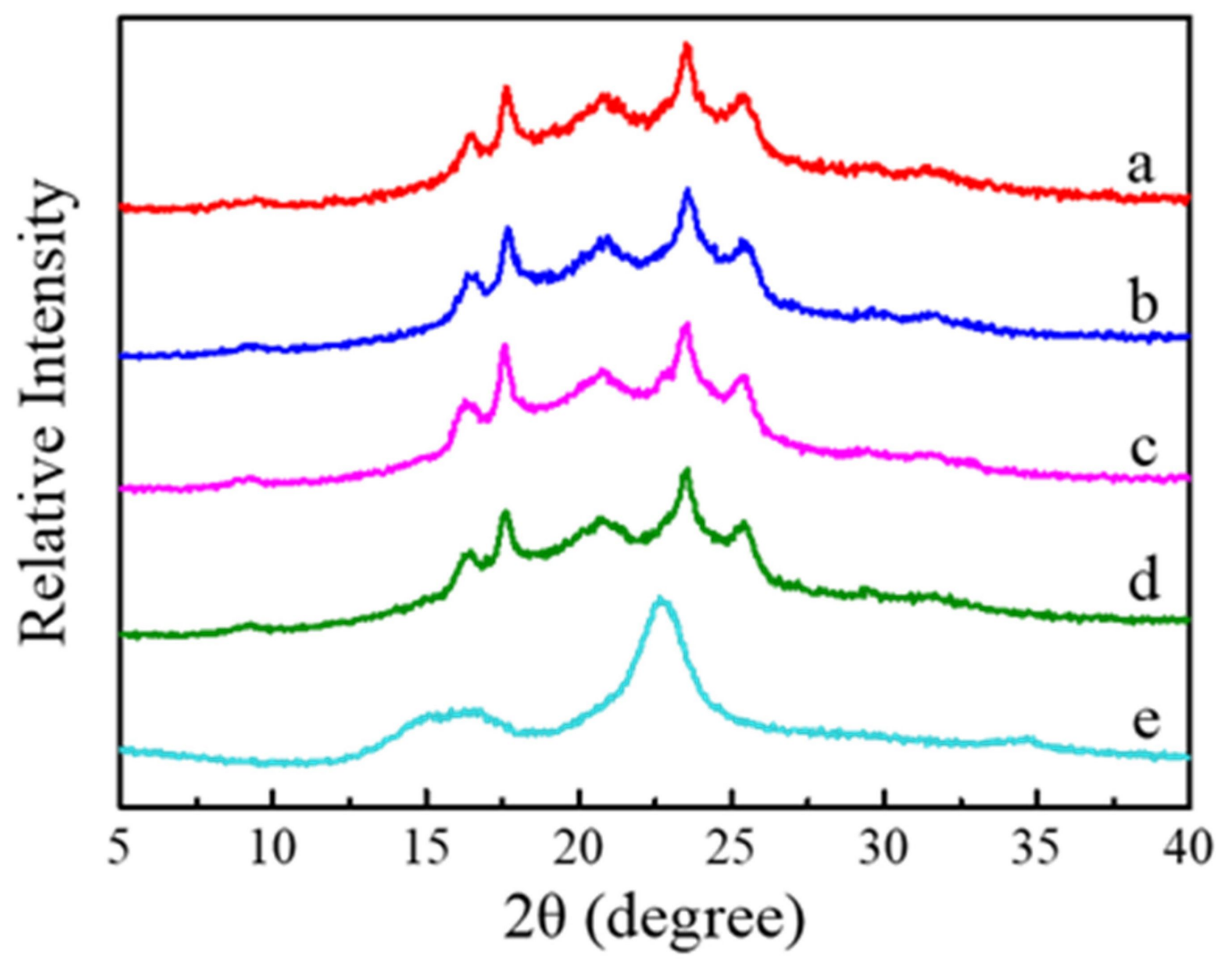
WAXD data for (**a**) g-PBAT-40, **(b**) 1 wt% g-PBAT-40/CNC, (**c**) 2 wt% g-PBAT-40/CNC, (**d**) 3 wt% g-PBAT-40/CNC, and (**e**) CNC samples.

**Figure 7:**
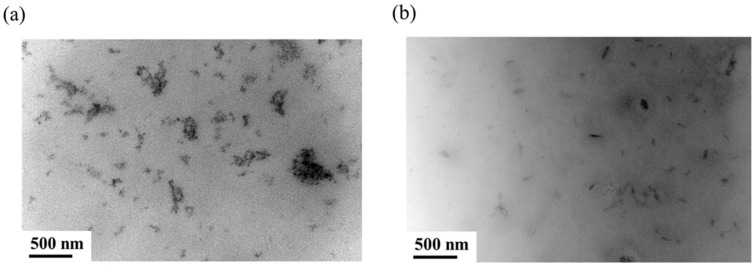
TEM images of 3 wt% (**a**) PBAT-40/CNC and (**b**) g-PBAT-40/CNC composites.

Figure 6 exhibits the WAXD diffraction profiles of the g-PBAT-40/CNC nanocomposites. For comparison, the X-ray diffraction profile of CNC is also shown in this figure. It is evident that no trace of diffraction peaks at 2θ = 14.9, 16.5, and 22.6° was obtained in the experimental results of the higher CNC-loading specimen, which is attributed to the well-dispersed CNC conformation. Similar results are also found for the g-PBAT-60/CNC and g-PBAT-80/CNC nanocomposites. Furthermore, the morphologies of 3 wt% g-PBAT-40/CNC nanocomposites are wholly examined using TEM. A TEM image of a 3 wt% g-PBAT-40/CNC nanocomposite is presented in Figure 7. This image discloses that the CNC is well distributed into the g-PBAT-40 copolyesters. For comparison, the TEM image of PBAT-40/CNC nanocomposite is also shown in this figure. It can be seen that the CNC is aggregated in PBAT-40 matrix. This result indicates that the grafting interaction of maleic acid onto the surface of PBAT-40 contributes to this result. Similar results are also found for the g-PBAT-60/CNC and g-PBAT-80/CNC nanocomposites. Consequently, the well-distributed morphologies of g-PBAT/CNC nanocomposites observed here through TEM images are in accordance with the WAXD diffraction data.

**Figure 8:**
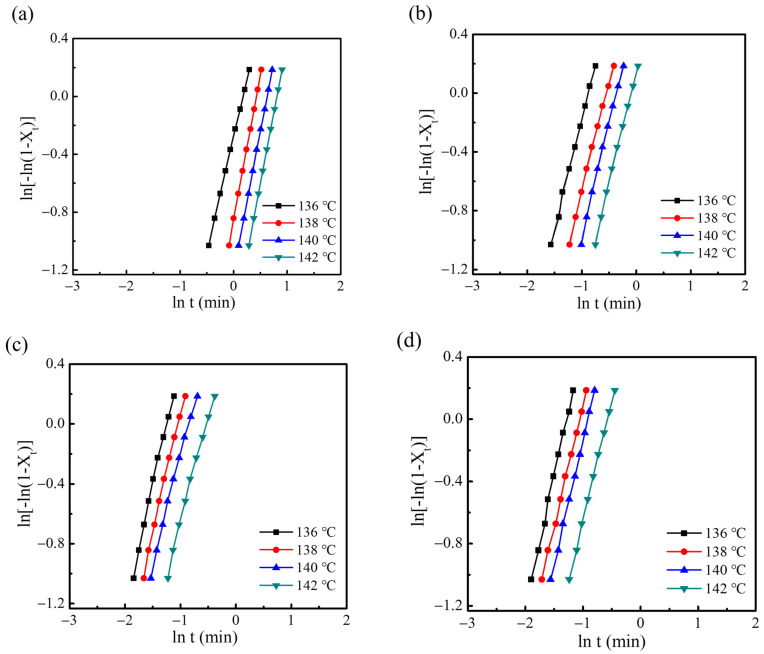
DSC data for (**a**) g-PBAT-40, (**b**) 1 wt% g-PBAT-40/CNC, (**c**) 2 wt% g-PBAT-40/CNC, and (**d**) 3 wt% g-PBAT-40/CNC nanocomposites.

Isothermal crystallization of g-PBAT matrix with different CNC contents were examined to study the influences of crystallization temperature (*T_c_*) on crystallization behavior of g-PBAT/CNC nanocomposites. The Avrami plots of g-PBAT/CNC nanocomposites are presented in Figure 8. All curves are approximately parallel to each other, revealing that the crystallization mechanism of the g-PBAT/CNC nanocomposites at different *T_cs_* remains the same. Table 3 summarizes the crystallization parameters, such as *n*-values, *k*-values, and *t*_1/2_, at various *T_cs_*. The n-value in the Avrami expression represents qualitative evidence on the mechanism of nucleation and the crystal growth formation. In Table 3, it can be seen that the n-values of g-PBAT40 are in the range from 1.60 to 2.0. The non-integral *n*-values suggest the occurrence of crystalline branching during crystallization as well as a mixed mechanism of nucleation and two-stage crystal growth [37,38]. Normally, the n-values near 2 are assigned to an athermal nucleation procedure followed by homogeneous nucleation and a two-dimensional crystal growth mechanism. The *n*-values of g-PBAT-40/CNC nanocomposites are in the range of 1.49–1.69, which makes them near to those of g-PBAT-40. Hence, these findings indicate that the crystallization mechanism of g-PBAT-40/CNC nanocomposites is similar to that of neat g-PBAT-40 polymer matrix. In addition, the *t*_1/2_ is used to examine the crystallization kinetics of g-PBAT-40/CNC nanocomposites. As shown in Table 3, *t*_1/2_ increases as Tc increases for all specimens, suggesting that the isothermal crystallization rate decreases as Tc increases. This behavior is attributed to the low supercooling at higher *T_c_*. By adding more CNC into g-PBAT-40/CNC up to 3 wt%, the *t*_1/2_ slightly decreases as the content of CNC increases. For example, the *t*_1/2_ values of g-PBAT-40 decrease remarkably from 0.95 to 0.22 min in nanocomposites with 3 wt% CNC contents when crystallized at the same crystallization temperatures. The half-time for crystallization of the 3 wt% g-PBAT/CNC composite decreased about 80% as compared with the same isothermal crystallization of neat polymer matrix. This implies that CNC accelerates the crystallization of g-PBAT-40 in the nanocomposites. Similar experimental tendencies for the g-PBAT-60/CNC and g-PBAT-80/CNC nanocomposites were obtained, and their crystallization parameters are summarized in Table 3.

**Figure 9:**
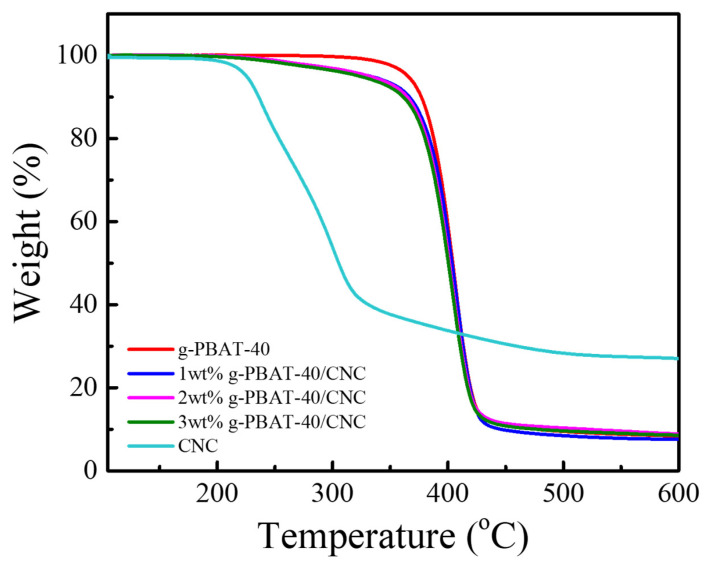
TGA profiles of PBAT-40, g-PBAT-40, CNC, and various weight ratios of g-PBAT-40/CNC nanocomposites.

In order to investigate the effect of CNC on the thermal behavior of g-PBAT/CNC nanocomposites, TGA analysis of g-PBAT matrix with various CNC content was performed. Figure 9 shows the TGA profiles of the g-PBAT-40/CNC nanocomposites. The experimental results for the g-PBAT-60/CNC and g-PBAT-80/CNC nanocomposites present related tendencies; the degradation temperatures found from these curves are listed in Table 4. The degradation temperature of 5 wt% weight loss for the neat g-PBAT-40 is slightly higher than those of g-PBAT-60 and g-PBAT-80. These studies reveal that g-PBAT-40 exhibits the best thermal stability among these synthesized biodegradable copolyesters. Nonetheless, all degradation temperatures of the g-PBAT/CNC nanocomposites decrease with increasing the CNC loadings. This occurrence is due to the existence of lower thermal stability CNC in the g-PBAT matrix, causing the decreasing thermal stability of nanocomposites. Similar results have been investigated in previous reports, for example, in the biodegradable ply(lactic acid)/layered double hydroxide and poly(butylene carbonate-co-terephthalate)/layered zinc phenylphosphonate nanocomposites [39,40].

**Figure 10:**
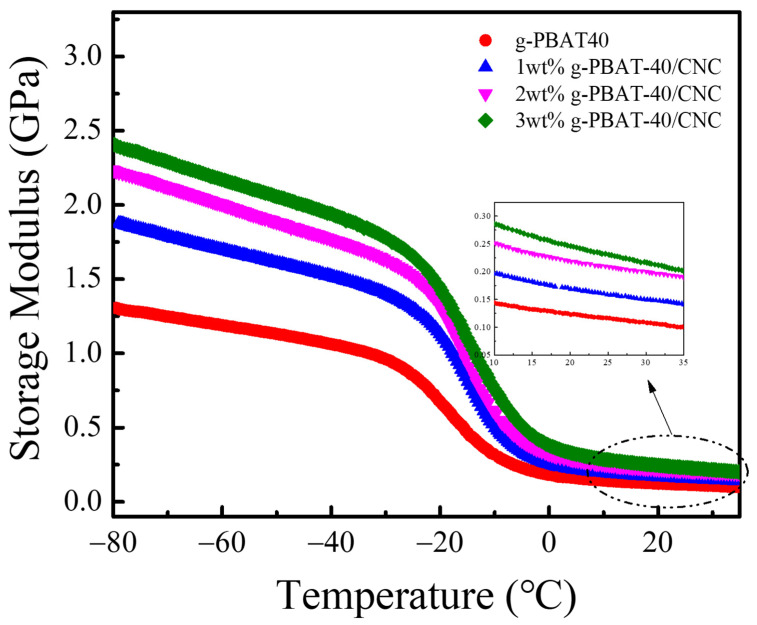
Dependence of the storage modulus on temperature of PBAT-40 and g-PBAT-40/CNC nanocomposites.

The storage modulus E’ against temperature of g-PBAT-40/CNC nanocomposites in a temperature range from −80 to 30 °C is presented in Figure 10. These findings reveal that the E’ of g-PBAT-40 is about 1.30 GPa at −80 °C and decreases with increasing temperature. These experimental data suggest that the molecular motion of g-PBAT at a temperature below the glass transition temperature *(T_g_*) is limited, whereas at a temperature higher than the *T_g_*, the thermal energy becomes comparable to the potential energy barriers of the molecular motions. The E’ of the g-PBAT-40/CNC nanocomposites at −80 °C is increased as the loading of CNC increases. Similar findings are also observed for the g-PBAT-60/CNC and g-PBAT-80/CNC nanocomposites. The E’ of all g-PBAT/CNC nanocomposites at 25 °C is also increased as the loading of CNC increases. Detailed E’ at −80 and 25 °C for all nanocomposites is also illustrated in Table 4. The improvement of E’ may be assigned to the reinforcement effect of the stiff CNC addition and its covalent linkages with g-PBAT, causing improvement in the rigidity of the g-PBAT polymer matrix. The glass transition temperatures (*T_gs_*) of all g-PBAT/CNC nanocomposites, also determined by DMA test, are increased as the loading of CNC increases. Detailed *T_g_* for all nanocomposites is also illustrated in Table 4. These results suggest that the incorporation of stiff CNC may inhibit the polymer chain motion, causing the increase in glass transition temperatures.

In order to understand the possible applications of the fabricated nanocomposites in food packaging, the water vapor barrier properties of g-PBAT/CNC nanocomposites were investigated. The WVP values of g-PBAT/CNC nanocomposites were summarized in Table 4. It can be seen that the WVP values of all g-PBAT/CNC nanocomposites are decreased with increasing loading of CNC. The decrease in WVP may be attributed to the addition of stiff CNC, causing the increase in the permeation route of the water molecules in the g-PBAT polymer matrix.

## 4. Conclusions

The new aliphatic–aromatic g-PBAT/CNC nanocomposites were fabricated using the transesterification and polycondensation process. FTIR and ^13^C-NMR spectra suggest the successful grafting of MA to PBAT. WAXD and TEM experimental results indicate that the CNC was randomly dispersed into the g-PBAT polymer matrix. The additional CNC in g-PBAT matrix enhanced the storage modulus as compared to that of neat g-PBAT. The decrease in the water vapor barrier properties of g-PBAT/CNC nanocomposites may be attributed to the addition of stiff CNC, causing the increase in the permeation route of the water molecules in the g-PBAT polymer matrix.

## Figures and Tables

**Table 1:** The synthesized polymer ratio, molecular weight, and crystallization temperatures of fabricated copolyesters.

Polymer	Feed Ratio [AA]/[DMT] (mol %)	Polymer Ratio ^a^ [AA]/[DMT] (mol %)	*M_w_* (g/mol) ×10^4^	*M_n_* (g/mol) ×10^4^	PDI	*T_c_* (°C)	*T_m_* (°C)
PBAT-40	40/60	39.5:60.5	3.91	2.31	1.69	118.1	154.5
PBAT-60	60/40	59.1:40.9	8.59	4.56	1.88	43.5	101.4
PBAT-80	80/20	80.5:19.5	10.47	5.60	1.87	3.70	42.1

^a^: Composition measured by ^1^H–NMR.

**Table 2:** Molecular weight and crystallization temperatures of grafted polyesters.

Polymer	*M_w_* (g/mol) × 10^4^	*M_n_* (g/mol) × 10^4^	PDI	*T_c_* (°C)	*T_m_* (°C)
g-PBAT-40	1.99	1.14	1.75	127.0	156.7
g-PBAT-60	2.36	1.21	1.94	64.3	106.4
g-PBAT-80	6.73	3.44	1.96	7.61	42.7

**Table 3:** Kinetic parameters of g-PBAT/CNC nanocomposites isothermally melt-crystallized at various *T_cs_*.

Sample	*T_c_* (°C)	*n*	*k* (min^−n^)	*t*_1/2_ (min)
g-PBAT-40	136	1.60	0.76	0.95
	138	2.00	0.43	1.27
	140	1.93	0.30	1.55
	142	1.96	0.21	1.86
1 wt% g-PBAT-40/CNC	136	1.49	3.71	0.32
	138	1.49	2.28	0.45
	140	1.56	1.78	0.55
	142	1.54	1.16	0.71
2 wt% g-PBAT-40/CNC	136	1.67	8.12	0.23
	138	1.61	5.44	0.28
	140	1.46	3.46	0.33
	142	1.42	2.14	0.45
3 wt% g-PBAT-40/CNC	136	1.69	8.80	0.22
	138	1.56	5.22	0.27
	140	1.61	4.35	0.32
	142	1.55	1.55	0.44
g-PBAT-60	82	2.03	2.53	0.53
	84	2.00	1.80	0.62
	86	1.91	1.24	0.74
	88	1.58	0.82	0.90
1 wt% g-PBAT-60/CNC	82	1.96	6.92	0.31
	84	1.98	5.60	0.35
	86	1.93	4.03	0.40
	88	1.90	2.76	0.48
2 wt% g-PBAT-60/CNC	82	2.06	12.7	0.24
	84	2.19	8.93	0.31
	86	1.92	4.07	0.40
	88	1.96	3.04	0.47
3 wt% g-PBAT-60/CNC	82	1.83	13.4	0.20
	84	1.85	9.67	0.24
	86	1.86	5.65	0.32
	88	1.78	4.05	0.37
g-PBAT-80	18	2.60	0.637	1.03
	20	2.72	0.178	1.65
	22	2.76	0.043	2.75
	24	2.79	0.009	4.80
1 wt% g-PBAT-80/CNC	18	2.66	0.701	1.00
	20	2.64	0.260	1.45
	22	2.79	0.074	2.23
	24	3.09	0.011	3.80
2 wt% g-PBAT-80/CNC	18	2.39	1.372	0.75
	20	2.60	0.393	1.24
	22	2.47	0.149	1.86
	24	2.68	0.024	3.51
3 wt% g-PBAT-80/CNC	18	2.86	2.311	0.66
	20	2.82	0.799	0.95
	22	2.99	0.250	1.41
	24	3.05	0.040	2.55

**Table 4:** Temperature of 5 wt% weight loss, temperature of the maximum degradation rate, storage modulus at −80 and 25 °C, Tg, and water vapor permeability (WVP) of the various g-PBAT/CNC nanocomposites.

Sample	^a^*T_5%_* (°C)	^b^*T_d_^max^* (°C)	*E’* at −80 °C (GPa)	*E’* at 25 °C (MPa)	*T_g_* (^o^C)	WVP (ng/m^2^·kPa·s)
g-PBAT-40	365.3	405.8	1.30	116.36	−11.36	124.1
1 wt% g-PBAT-40/CNC	332.6	404.4	1.87	158.01	−9.17	82.7
2 wt% g-PBAT-40/CNC	332.0	403.9	2.24	208.64	−9.01	65.4
3 wt% g-PBAT-40/CNC	323.3	403.6	2.41	230.43	−7.63	48.9
g-PBAT-60	355.8	409.5	0.94	39.02	−28.97	157.9
1 wt% g-PBAT-60/CNC	322.2	409.7	1.57	65.38	−27.47	136.8
2 wt% g-PBAT-60/CNC	307.3	409.0	1.75	71.16	−25.91	91.7
3 wt% g-PBAT-60/CNC	291.0	405.4	2.15	74.30	−25.33	77.4
g-PBAT-80	335.8	413.3	1.59	130.28	−31.59	144.4
1 wt% g-PBAT-80/CNC	318.4	412.5	2.21	174.52	−27.75	91.7
2 wt% g-PBAT-80/CNC	295.1	412.1	2.48	203.14	−27.33	73.7
3 wt% g-PBAT-80/CNC	278.2	411.8	2.96	222.14	−27.19	65.2

^a^*T_5%_*: Temperature of 5 wt% weight loss. ^b^*T_d_^max^*: Temperature of the maximum degradation rate.

## Data Availability

The data presented in this study are available on request from the corresponding author.
